# Using artificial intelligence to study atherosclerosis from computed tomography imaging: A state-of-the-art review of the current literature

**DOI:** 10.1016/j.atherosclerosis.2024.117580

**Published:** 2024-11

**Authors:** Laura Valentina Klüner, Kenneth Chan, Charalambos Antoniades

**Affiliations:** Acute Multidisciplinary Imaging and Interventional Centre, Division of Cardiovascular Medicine, Radcliffe Department of Medicine, Oxford NIHR Biomedical Research Centre, University of Oxford, United Kingdom

**Keywords:** AI, Atherosclerosis, CCTA, CT, Coronary artery disease, EAT, FAI, Fat attenuation index, FRP, HRP, Imaging, Inflammation, ORFAN, Plaques, Radiomics, Radiotranscriptomics

## Abstract

With the enormous progress in the field of cardiovascular imaging in recent years, computed tomography (CT) has become readily available to phenotype atherosclerotic coronary artery disease. New analytical methods using artificial intelligence (AI) enable the analysis of complex phenotypic information of atherosclerotic plaques. In particular, deep learning-based approaches using convolutional neural networks (CNNs) facilitate tasks such as lesion detection, segmentation, and classification. New radiotranscriptomic techniques even capture underlying bio-histochemical processes through higher-order structural analysis of voxels on CT images. In the near future, the international large-scale Oxford Risk Factors And Non-invasive Imaging (ORFAN) study will provide a powerful platform for testing and validating prognostic AI-based models. The goal is the transition of these new approaches from research settings into a clinical workflow.

In this review, we present an overview of existing AI-based techniques with focus on imaging biomarkers to determine the degree of coronary inflammation, coronary plaques, and the associated risk. Further, current limitations using AI-based approaches as well as the priorities to address these challenges will be discussed. This will pave the way for an AI-enabled risk assessment tool to detect vulnerable atherosclerotic plaques and to guide treatment strategies for patients.

## Highlights

• There is a shift from qualitative plaque characterization to advanced plaque phenotyping using artificial intelligence.
• Measuring plaque inflammatory burden is possible by using the fat attenuation index (FAI) score and radiotranscriptomics.
• The integration of multidimensional data into AI-based risk prediction models allows personalized patient management.

## Graphical abstract

**Figure:**
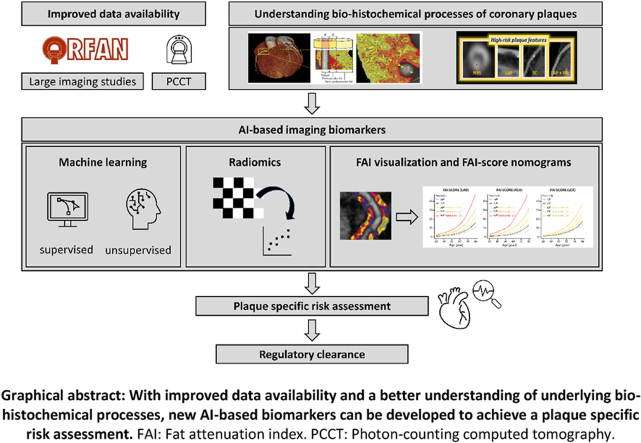

## 1 Introduction

Cardiovascular diseases (CVDs) remain the leading cause of death worldwide with atherosclerotic coronary heart disease being the most common CVD [1]. It is recognized that atherosclerosis is not simply the accumulation of low-density lipids (LDLs) in the arterial intima, but that the infiltration of LDL leads to inflammation which mediates all stages of atherogenesis. Monocytes circulate along the activated endothelial layer that expresses adhesion molecules such as vascular-cell adhesion molecule 1 (VCAM-1). Once migrated into the intima, proinflammatory cytokines and macrophage colony-stimulating factors (M-CSF) induce monocyte differentiation into macrophages that scavenge oxidized LDL. T-lymphocytes and macrophages are involved in the process of atherosclerotic plaque formation by promoting smooth muscle cell migration to form a fibrous cap around the lipid-rich necrotic core. Acute plaque rupture occurs as inflammation mediated collagenases expression alters the extracellular matrix of the fibrous cap, ultimately triggering thrombosis that leads to an occlusion of the coronary artery [2,3]. Early clinical studies have demonstrated that increased inflammation, measured by inflammatory markers such as C-reactive protein, is associated with worse cardiovascular outcomes [4]. However, these systemic markers are not necessarily cardiovascular specific. Translational studies have discovered that phenotypic changes of adipocytes in the perivascular space in response to inflammation are non-invasively detectable on computed tomography (CT) imaging [5]. The usage of AI-based analysis of perivascular adipose tissue (PVAT) on routinely performed coronary CT angiography (CCTA) offers new opportunities to assess coronary inflammation in addition to quantitative analysis of atherosclerotic plaques [6].

In this review, we discuss the applications of AI in cardiovascular imaging, in particular automated segmentations of anatomical structures of the heart as well as coronary plaque segmentations, characterization, and prognostic modelling. Finally, we highlight the current evidence gap and limitations of these techniques and discuss the perspectives for future research (Graphical abstract).

## 2 Imaging of coronary plaque characteristics

CCTA is recommended by clinical guidelines as the first-line non-invasive investigation for patients with suspected coronary artery disease (CAD) [[7], [8], [9]]. The current interpretation of CCTA images focuses on the degree of coronary stenoses that guide interventional angioplasty to revascularize luminal obstructions. However, insights from the ISCHAEMIA trial have demonstrated that this approach did not significantly improve cardiovascular outcomes in the context of stable coronary disease [10]. Indeed, about 50 % of the acute coronary syndrome (ACS) events occur due to rupture of non-obstructive plaques. The importance of plaque characterization beyond luminal stenosis is increasingly recognized in efforts to capture vulnerable plaques that are at risk to rupture, and to guide early preventative therapy [11].

The coronary artery calcium (CAC) score captures the calcified burden of coronary plaques. Derived from non-contrast CT scans, it is a metric that takes into account the volume of calcified components as well as their density measured by attenuation values on CT to form a risk score. However, the application of the CAC score is largely limited to risk stratification in the asymptomatic population [12]. Statin therapy itself leads to calcification and stabilization of atherosclerotic plaques, resulting in an increase in CAC score [13]. Further, the CAC score fails to capture the risk of non-calcified plaques that are prone to rupture, thus limiting its prognostic value for ACS prediction.

**Fig. 1:**
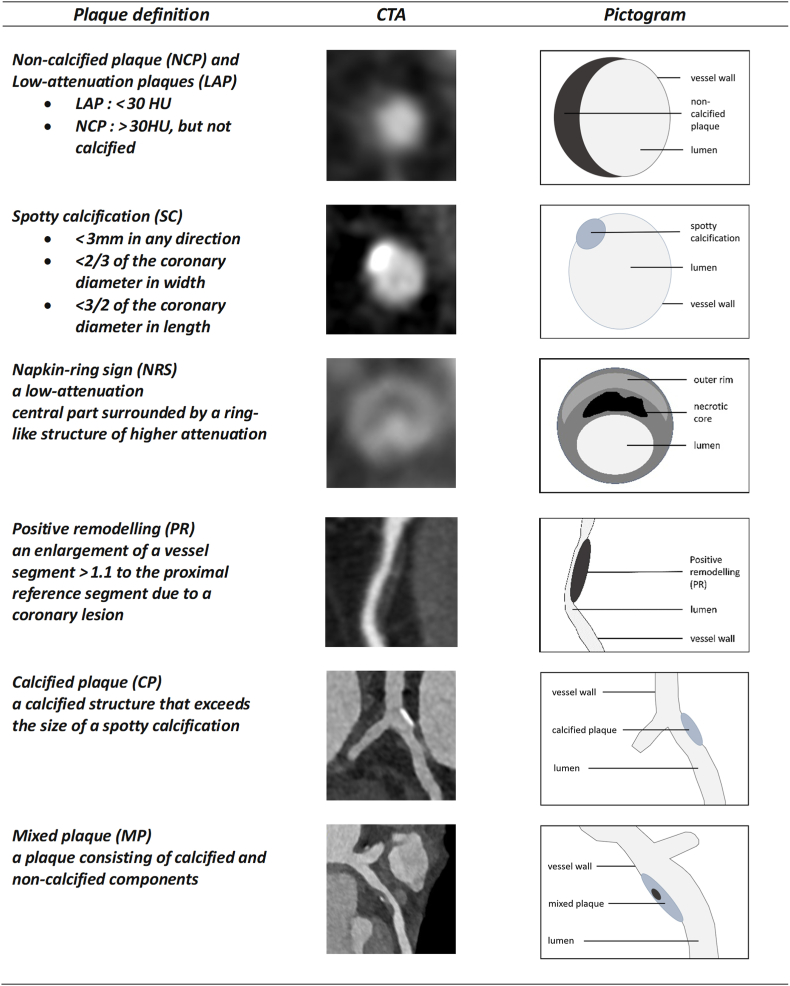
Overview of plaque definitions and appearance on CTA. CP: calcified plaque. LAP: low-attenuation plaque. HU: Hounsfield unit. NCP: non-calcified plaque. MP: mixed plaque. NRS: Napkin-ring sign. PR: positive remodelling. SC: spotty calcification.

In the classification of coronary plaques on CCTA different plaque types can be further distinguished based on their composition or visual imaging patterns. With respect to the analysis of coronary plaques compared to a purely clinical risk assessment, quantitative atherosclerosis characterization is superior to clinical/laboratory variables or qualitative plaque evaluation to predict the risk of coronary plaque progression [14]. Six main plaque types are commonly described: calcified plaques (CP), mixed plaques (MP), non-calcified plaques (NCP) (with low-attenuation plaques (LAP) as a subgroup), the Napkin-ring sign (NRS), positive remodelling (PR), and spotty calcification (SC). The latter four plaque types (LAP, NRS, PR and SC are recognized as high-risk plaque (HRP) features that were shown to predict ACS independent of significant stenosis [11] (Fig. 1). In addition, non-ST-elevation myocardial infarction (NSTEMI) and unstable angina pectoris (UA) were found to occur more frequently in segments with non-calcified plaques compared to those with calcified plaques [15]. The density of calcified plaques is also a crucial determinant of the risk of ACS, where dense calcified plaques with an attenuation of more than 1000 Hounsfield units (HU) on CT are associated with a lower risk compared to those with a lower attenuation [16]. For spotty calcifications, it was demonstrated that these were larger in number, smaller in size, shallower, and closer to the plaque rupture site in patients with ACS [17]. The group of non-calcified plaques can be divided into fibrous plaques and lipid-rich plaques, the latter being a subgroup referred to as low-attenuation plaques (<30 HU on CCTA) which became the focus of various studies. In contrast to fibrous plaques, LAPs contain a lipid core, in which the risk of myocardial infarction (MI) is up to 5 times higher when the LAP burden is greater than 4 % [18]. Despite these findings, the value of LAPs remains controversial as the characterization is prone to technical variabilities, and the area of low-attenuation from artefacts such as beam hardening affects the quantification [19,20]**.**

In addition, other limitations regarding the coronary plaque classification and its prognostic value remain. First, despite numerous studies that demonstrated the impact on the predictability of myocardial infarction, heterogenous definitions were used for the same plaque types. For example, various cut-off values for HUs were applied to differentiate low-attenuation from non-calcified values, and similarly from calcified plaques **(**Table 1 and Table 2). Secondly, Channon et al. [21] pointed out that only 24 of 1019 HRPs resulted in non-fatal myocardial infarction in the PROMISE study [22]. Conversely, in the SCOT-HEART trial >30 % of cardiac events occurred in patients who did not have HRPs at all [23]. This highlights the pitfall of using high-risk plaques alone in cardiovascular risk stratification. Therefore, there is the unmet need for refined imaging biomarkers that objectively define vulnerable plaques which are at risk of causing acute cardiovascular events.

**Table 1:** Discrepancies in the definitions of different types of non-HRP features.

Author	Year	Characteristics
**Definitions of mixed plaques in the literature**		
Leber et al. [24]	2003	They only considered plaques as mixed plaques if > 50 % of the voxels were non-calcified. Their rational for this cut-off was due to potential blooming artefacts caused by large calcifications.
Maurovich-Horvat et al. [25]	2012	They simply defined mixed plaques as any plaque containing both calcified and non-calcified voxels.
Feuchtner et al. [26]	2017	They subdivided mixed plaques into the two categories: dominantly calcified and dominantly non-calcified.

**Definitions of calcified plaques in the literature**		
Achenbach et al. [27]	2004	Calcified plaque: ≥ 130 HU
Motoyama et al. [28]	2007	Calcification was defined to be > 220 HU
Brodoefel et al. [29]	2008	Calcified plaque: ≥ 437 HU, using intravascular ultrasound – virtual histology (IVUS-VH) as a reference for their threshold
Inoue et al. [30]	2010	Calcified plaque: 350–1000 HU
Obaid et al. [31]	2013	Calcified plaque: > 465 HU in correlation with IVUS-VH
Oikonomou et al. [32]	2018	Calcified plaque: > 465 HU, referring to Obaid et al. [31]
Van Rosendael et al. [16]	2020	Calcified plaque: ≥ 350 HU
Lee et al. [33]	2021	Calcified plaque: ≥ 351 HU
Jávorszk et al. [34]	2022	Calcified plaque: ≥ 351 HU, referring to Inoue et al. [30]

HU: Hounsfield unit. IVUS-VH: intravascular ultrasound virtual histology.

**Table 2:** Discrepancies in the definitions of different types of HRP features.

Author	Year	Characteristics
**Definitions of spotty calcifications in the literature**		
Kajinami et al. [35]	1997	SCs are < 2/3 of the coronary diameter in width and < 3/2 of the coronary diameter in length.
Ehara et al. [36]	2004	Through an IVUS study comparing patients with acute coronary syndrome (ACS) and patient with stable angina pectoris (AP), SC was defined to have an arc < 90°.
Kitagawa et al. [37]	2009	Referring to the definition of Kajinami et al. [35]
Motoyama et al. [38]	2009	SC defined to be < 3 mm in size.
Van Velzen et al. [39]	2011	In addition to the definition of Kajinami et al. [35], they further subdivided SC into small spotty calcification (< 1 mm), intermediate spotty calcification (1–3 mm), and large spotty calcification (≥ 3 mm).
Puchner et al. [11]	2014	In addition to the definition of Kajinami et al. [35], they defined SC to be < 3 mm in any direction.
Kataoka et al. [40]	2014	SC defined to be < 4 mm in size and a maximal arc < 90° on optical coherence tomography (OCT).
Ong et al. [41]	2016	Referring to the definition of Kataoka et al. [40]
Obaid et al. [42]	2017	SC defined to be < 3 mm in size.

**Definitions of NRS in the literature**		
Tanaka et al. [43]	2001	They defined the ring of the NRS to be < 130 HU.
Kashiwagi et al. [44]	2009	They referred to the definition by Tanaka et al. [43]
Maurovich-Horvat et al. [45]	2010	Even though they agreed on the general appearance with a low-attenuation central part surrounded by a ring-like structure of higher attenuation, they did not specify an upper threshold for the voxels inside the ring.
Maurovich-Horvat et al. [25]	2012	No upper threshold for voxels inside the ring was used.
Seifarth et al. [46]	2012	They showed that the median density in the central area was 48.1 HU while having 68.2 HU for the median density in the rim of the NRS. They further discussed the range of attenuation values. They referred it to partial volume effects, and also highlighted the necessity for imaging markers that not only rely on attenuation values.
Otsuka et al. [47]	2013	They aligned with the definition by Tanaka et al. [43]
Kolossváry et al. [48]	2017	They used the definition by Maurovich-Horvat et al. [45] and did not define a maximum threshold for the ring.
Feuchtner et al. [26]	2017	They defined the ring to be < 200 HU and the hypodense core to be < 130 HU.

**Definitions of non-calcified plaques and low-attenuation plaques in the literature**		
Schroeder et al. [49]	2001	A differentiation between soft and intermediate plaques was pursued (intracoronary ultrasound (ICUS) against CT). According to Schroeder et al., soft plaques have a density < 50 HU while intermediate plaques lie between 50 HU and 119 HU.
Becker et al. [50]	2003	The attenuation of lipid-rich plaques (47 ± 9 HU) was significantly different from fibrous plaques (104 ± 28 HU), analyzed in CT vs histopathology.
Achenbach et al. [27]	2004	They defined a NCPs as any structure inside the coronary wall that has a lower attenuation than the contrast in the lumen, but a higher attenuation than the surrounding tissue.
Leber et al. [24,51]	2003, 2004	They referred to the definition of Schroeder et al. [49]
Carrascosa et al. [52]	2006	They defined non-calcified plaques to be < 185 HU. A threshold of 88 HU served as the differentiation between soft plaques and fibrous plaques, analyzed in CT vs IVUS.
Hoffmann et al. [53]	2006	They referred to Schroeder et al. [78] and Achenbach et al. [27]
Motoyoma et al. [28]	2007	They first divided NCPs into the two categories: NCP < 30 HU and 30 HU < NCP < 150 HU. These cut-offs were derived by comparison between CCTA measurements and IVUS data of the same patients.
Sun et al. [54]	2008	The mean density of soft plaques was 72 HU while for fibrous plaques it was 90 HU. The mean attenuation in the lumen was 398 ± 74 HU in their study, analyzed in CT vs IVUS.
Brodoefel et al. [29]	2008	Fatty plaques: 10HU to 69 HU; Fibrous plaques: 70 HU to 158 HU; using IVUS-VH as a reference for their thresholds.
Inoue et al. [30]	2010	NCP: 100 HU to 350 HU; LAP < 30 HU; referring to Motoyama et al. [38]
Dalager et al. [55]	2011	They defined lipid-rich plaques with HU-values < 60 HU while fibrotic plaques were considered to be in the range between 61 HU and 119 HU, analyzed on CT vs IVUS.
Marwan et al. [56]	2011	They referred to the definition of Motoyama et al. [28], but investigated that a threshold of 5.5 % of voxels < 30 HU revealed the best sensitivity and specificity to identify lipid-rich plaques. They analyzed CT vs IVUS.
Ferencik et al. [57]	2012	They tested various thresholds for NCPs and found that < 90 HU was more predictive for ACS than the threshold of 30 HU proposed by Motoyama et al. [28]
Maurovich-Horvat et al. [25]	2012	They referred to the definition of NCPs by Achenbach et al. [27]
Obaid et al. [31]	2013	Necrotic core: 1 HU to 64 HU; Fibrous plaques: 65 HU – 260 HU in correlation with IVUS-VH. CTs in this study had a median lumen attenuation of 362 HU.
Schlett et al. [58]	2013	They defined LAPs by < 90 HU when comparing CT vs histology of five ex vivo hearts.
Schlett et al. [59]	2013	In a comparison with histological data, they found out that LAPs < 60 HU showed the strongest correlation with histological confirmed lipids.
Feuchtner et al. [26]	2017	They defined LAPs to be < 60 HU
Van Rosendael et al. [16]	2020	LAP < 30 HU
Jávorszk et al. [34]	2022	NCP: -100 to 350 HU, referring to Inoue et al. [30]

**Definitions of positive remodelling in the literature**		
Schoenhagen et al. [60]	2000	PR ≥ 1.05, based on Pasterkamp et al. [61] who performed analysis on femoral arteries.
Imazeki et al. [62]	2004	PR ≥ 1.10
Raffel et al. [63]	2008	PR ≥ 1.05, referring to Schoenhagen et al. [60]
Motoyama et al. [38]	2009	PR ≥ 1.10
Gauss et al. [64]	2011	PR ≥ 1.10 showed better results than PR ≥ 1.05 in comparison with IVUS.
Conte et al. [65]	2017	PR ≥ 1.10

ACS: acute coronary syndrome. AP: angina pectoris. CCTA: coronary computed tomography angiography. CT: computed tomography. HU: Hounsfield unit. IVUS: intravascular ultrasound. LAP: low-attenuation plaque. NCP: non-calcified plaque. NRS: Napkin-ring sign. OCT: PR: positive remodelling. SC: spotty calcification.

## 3 AI for cardiovascular imaging

The key for an accurate risk prediction of atherosclerotic coronary disease is to reveal the underlying histological and biochemical processes that are detectable by non-invasive imaging and to incorporate those into predictive algorithms. AI is a useful tool to assist in the analysis of a huge amount of input data. AI represents the umbrella term for all kinds of computational processes which mimic human intelligence. Machine learning (ML), a subset of AI, is the process of machines learning through experience [66]. In general, the field of ML can be subdivided into supervised and unsupervised ML. While the first requires labeled datasets for the prediction of a particular outcome class, the latter reveals unknown patterns in unlabeled data [66]. Deep learning (DL) represents a subset of ML which uses algorithmic structures imitating neural networks of the human brain. A specific type of these DL-networks are the convolutional neural networks (CNNs), which are widely used in the field of biomedical imaging to perform tasks such as lesion detection, segmentation, classification, reconstruction, and regression [67,68].

### 3.1 AI applications for automated segmentation of the cardiac structures

Segmentation of anatomical structures from cardiac imaging is the key step for quantitative analysis. Automated segmentations are desirable to enable high-throughput analysis of a large volume of imaging data. Based on the manually segmented and annotated ‘ground truth’ image, the input image is encoded into low-resolution maps for learning useful features and up-sampled to full resolution of predicted segmentations through the DL-network [68]. The performance is then evaluated by metrics such as the Dice coefficient or Jaccard score to assess the regional overlap between the predicted and the ground truth segmentation [69]. Further fine-tuning could be achieved through methods such as reinforcement learning in which the model learns through experience from feedback loops [70]. Particularly CNN models have been successfully trained to segment anatomical structures such as cardiac chambers [71,72] and epicardial adipose tissue (EAT) [70,73] that facilitated the development of prognostic models to predict cardiovascular risk.

### 3.2 AI applications for coronary trees and plaques

DL-models could streamline the workflow in coronary analysis and assist with the quantitative evaluation of atherosclerotic plaques. Early semi-automatic approaches showed meaningful proof of feasibility for DL-based segmentation techniques of coronary plaques, although the training data was small and required time-consuming user interaction [74,75]. Recently, two promising DL-based research applications were built. Lin et al. [76] trained a hierarchical convolutional long short-term memory (ConvLSTM) network using 5045 lesions from 921 CCTA scans achieving a good interclass correlation to an expert reader. Jávorszky et al. [34] developed a 3D U-net model which achieved interclass correlation (ICC) values of 0.95 in CPs, 0.93 in NCPs, 0.86 in LAPs and 0.94 in total plaque volume. These results indicate the potential of DL-based segmentations in atherosclerotic disease. Indeed, a plethora of commercially available software applications (e.g. Autoplaque, CaRi-Heart, Cleerly, Heartflow Plaque Analysis) are already available to integrate DL-based segmentations of the vascular wall to facilitate atherosclerotic plaque analysis. However, two major challenges remain. First, these DL-models are largely based on classifying atherosclerotic plaques quantifying the components (calcified, non-calcified, low-attenuation), which are not necessarily trained against hard cardiovascular outcomes of acute plaque rupture. Secondly, other factors influencing the vulnerability of individual plaques such as inflammation are yet to be integrated.

### 3.3 AI applications on coronary inflammation

**Fig. 2:**
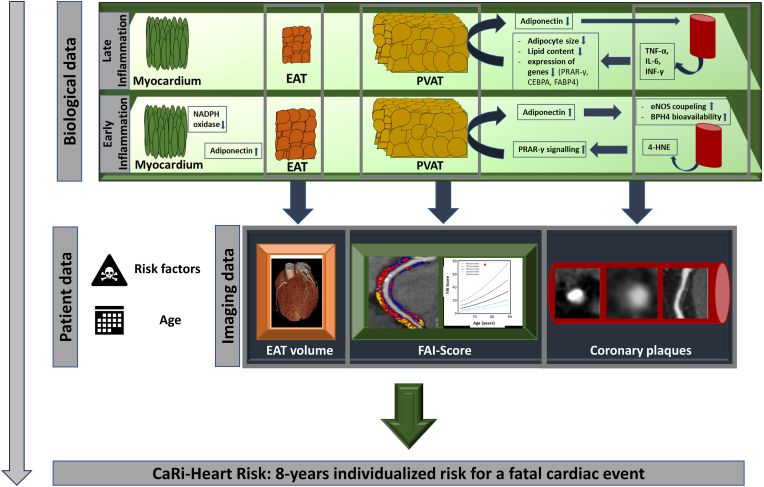
Using the knowledge of underlying biochemical processes of early and late inflammation, imaging biomarkers can be developed and incorporated into the CaRi-Heart Risk score for fatal cardiac events. 4-HNE: 4-hydroxynonenal, CEBPA: CAAT/enhancer-binding protein alpha, EAT: epicardial adipose tissue, eNOS: endothelial nitric oxide synthase, FABP4: fatty acid-binding protein 4, FAI: fat attenuation index, IL-6: Interleukin-6, INF-γ: Interferon‐γ, NADPH: Nicotinamide Adenine Dinucleotide Phosphate Hydrogen, PRAR-γ: peroxisome proliferator-activated receptor γ, PVAT: perivascular adipose tissue, TNF-α: tumor necrosis factor α.

In 2013 Margaritis et al. [77] described the crosstalk between pericoronary fat (PVAT) and the coronary wall in a bidirectional way through paracrine signaling. Subsequent studies explored the complex relationship of gene expression and cytokines released by both the coronary wall and PVAT [[77], [78], [79]] (Fig. 2). It was shown that coronary inflammation leads to lipolysis and smaller sized adipocytes in PVAT, and therefore a shift from a lipid to a more aqueous phase. These findings formed the basis for the development of the CCTA imaging biomarker fat attenuation index (FAI), which is widely recognized as an imaging biomarker of vascular inflammation derived from CCTA. Indeed, FAI captures the three-dimensional gradients of perivascular attenuation signals within the adipose tissue window (-30 to -190 HU) after corrections for several anatomical and technical parameters; its ability to reflect vascular inflammation has been validated against histological biopsies by using rigorous translational science methodologies [5]. The landmark CRISP-CT study has shown two key findings [32]: First, individuals with elevated inflammation (FAI ≥ -70.1 HU in the CRISP-CT population) were found to have a higher risk of all-cause and cardiac mortality; secondly, FAI values were modifiable by anti-inflammatory therapy such as statins. This was further confirmed in a cohort of psoriasis patients in whom FAI was reduced one year after treatment with biologic agents [80]. Therefore, FAI showed to be the first imaging biomarker capturing early coronary inflammation on CT imaging.

It is important to note that PVAT FAI values are uncorrected measurements and could be influenced by technical, anatomical, and biological factors. Indeed, unadjusted PVAT FAI measurements (or pericoronary adipose tissue attenuation – PCAT) were found to be less predictive for cardiovascular outcomes compared with weighted FAI measurements as discussed in a recent ESC Clinical Consensus Statement [81]. FAI-Score was developed as a standardized algorithm to incorporate in addition to FAI, further anatomical, biological and technical factors related to the image acquisition, as well as the age and sex of the individual. The standardized metric, calculated for the left anterior descending (LAD), left circumflex artery (LCX) and right coronary artery (RCA), is projected on age- and sex-specific nomograms for clinical interpretation of coronary inflammation. FAI-Scores above the 75th percentile in the LAD and RCA and above the 95th percentile in the LCX were shown to have a 2.4 times higher risk of fatal cardiac events, whereas FAI-Scores above the 90th percentile in the LAD and RCA are considered to be very-high risk, with a relative risk > 3 and > 5 respectively of fatal cardiac events in the CRISP-CT study [82].

### 3.4 AI application integrating radiomics

Further imaging biomarkers could be extracted by analyzing radiomic data using DL-methods. Radiomics capture structural relationships of voxels, i.e. the smallest 3D-volumes measured on CT images, which are not visible to the naked human eye. Radiomic features are derived from imaging data to provide a higher-level synthesis of quantitative metrics such as intensity, shape, and texture [83]. They can also be analyzed in conjunction with other -omics data to answer biologically relevant questions. For example, radiotranscriptomics combine quantitative imaging data with the gene expression state of tissue derived from laboratory experiments. This means that different gene expression states can be correlated with imaging by applying structural analysis of voxels on CT data [84,85]. This is achieved by defining the molecular ground truth of the tissue of interest using RNA sequencing/gene expression data derived from biopsies of that tissue, and then training a radiomic signature of the image of that tissue against the constructed transcriptomic ground truth [84,85]. In general, these parameters can be subdivided into four categories: intensity-based metrics (first-order statistics), texture-based metrics (second- and higher-order statistics), shape-based metrics and transformation-based metrics [83].

Applications of radiomics in the field of CVDs range from myocardial scar detection [86] to coronary plaque analysis and prognostication. Recently, radiomics were applied in patients with COVID-19 developing the radiotranscriptomic signature C19-RS, which was trained on imaging data from routine CT pulmonary angiography scans and linked with transcriptomic data of cytokine-driven inflammation of internal mammary artery biopsies. C19-RS was predictive for in-hospital mortality and could thus guide treatment procedures [85].

In terms of atherosclerotic CVD, the first study using radiomics for coronary plaque classification was performed in 2017. The radiomic signature was identified by analyzing 916 radiomic variables of just 30 plaques with NRS and 30 with non-NRS of similar degree of calcification [48]. Compared to just histogram-based analysis of voxels (first-order statistics), radiomic analyses improved the identification of advanced coronary plaque lesions [87]. When examining 25 coronary plaques using CCTA, NaF^18^-PET, IVUS and OCT, it was shown that radiomics have the potential to capture biomarkers from invasive or radionuclide imaging [88]. In 2021, radiomics were applied to differentiate between stable and unstable coronary plaques in a study comprising 60 patients with an acute myocardial infarction against 60 patients with stable CAD. Lin et al. [89] extracted radiomic variables which were specific for both culprit and high-grade stenosis non-culprit lesions.

Apart from applying radiomics to coronary plaques themselves, a radiomic signature for PVAT, namely the fat radiomic profile (FRP) has been developed [84]. In contrast to FAI that quantifies the dynamic changes due to acute inflammation, FRP also captures the structural changes in PVAT by linking the gene expression state for inflammation (expression of TNF-alpha), angiogenesis (expression of CD31) and fibrosis (expression of COL1A1) with radiomic variables derived from imaging data to generate the radiotranscriptomic signature. In validation studies, the improvement in the predictive value for MACE was proven to be beyond traditional risk factors, the CAC score, the degree of stenosis or HRP features on CCTA [84]. Several studies in various settings and patient numbers have shown the feasibility of radiomic signatures to reveal complex patterns in the data by AI-approaches. In the near future, risk profiling of individual atherosclerotic plaques will be achievable by integrating radiomic approaches into applications for clinical practice.

## 4 AI for predicting cardiovascular risk

Apart from automated segmentation and classification of imaging data, AI-approaches can be used to integrate emerging imaging biomarkers and known traditional risk factors into cardiovascular risk prediction. For example, the standardized FAI-Score metric is incorporated into a risk prediction algorithm along with variables such as demographics (age and sex) and known cardiovascular risk factors (hypertension, diabetes, smoking status, hyperlipidemia) to provide an absolute 8-year risk (CaRi-Heart Risk) of fatal cardiac events [82]. This allows further prognostic information from routinely performed CCTA scans for patients with suspected coronary artery disease. It provides a more accurate risk prediction in symptomatic patients than current risk scores which are dependent on clinical risk factors alone and were originally developed for primary cardiovascular prevention in the general population.

**Fig. 3:**
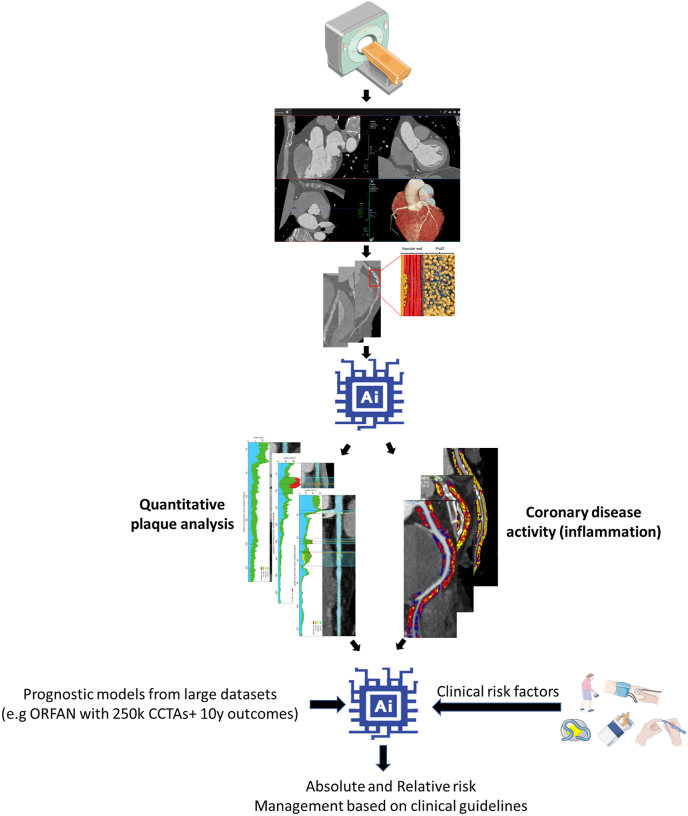
Artificial intelligence-assisted image interpretation for the assessment of coronary artery disease, prediction of patient's risk, and development of personalized medical management. Reproduced with permission from Antoniades et al. [6].

The prospect of large-scale international cohorts such as the Oxford Risk Factor and Non-invasive Imaging (ORFAN) study, in which CCTA imaging is linked with long-term clinical outcomes, offers opportunities to train new imaging biomarkers against hard outcomes. By including actual ACS events over a follow-up period, a deep learning time-to-event model would allow training of a prognostic model that predicts the vulnerability of atherosclerotic plaques. At the patient level, such AI-based models could be combined with non-imaging biomarkers such as genetic determinants, as well as non-imaging biomarkers from clinical information to detect individual patients with polygenic predisposition of cardiovascular events. This would create a comprehensive risk assessment to highlight vulnerable patients and to guide personalized treatment strategies by modifying individual risk factors of patients (Fig. 3).

## 5 AI in the assessment of atherosclerosis: challenges and opportunities

**Fig. 4:**
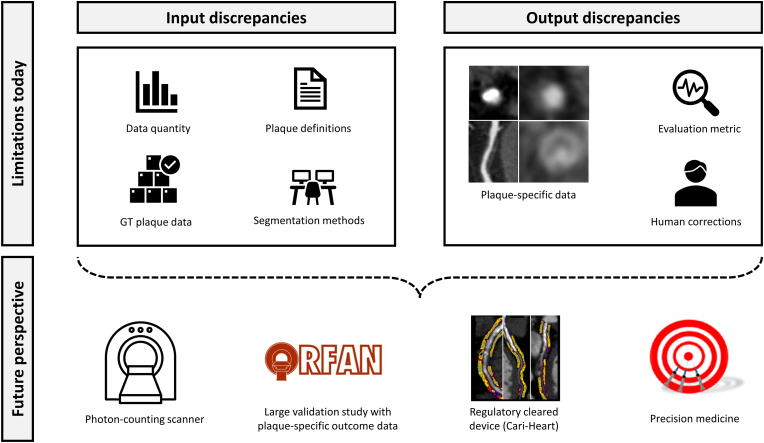
What we understand, what we are able to address with AI, and what is missing.

Besides the enormous progress in the integration of AI-based approaches to study atherosclerosis, there are remaining challenges which need to be addressed. In general, the application of AI in atherosclerotic diseases is still in the sphere of a “black box” concept which might prevent clinicians from incorporating these tools into clinical workflows. While the usage of AI tools in comprehensible segmentation tasks has been applied more frequently, complex models for risk prediction are still viewed with restraint [90]. To improve this perception, further challenges need to be tackled in the first instance. They can be divided into remaining challenges regarding the input and the output data (Fig. 4). In the following, these will be outlined separately for the application of AI-based tools to assess coronary plaques.

Regarding the input discrepancies, both data quantity and data quality need to be reviewed. In terms of data quantity, there is a striking need for large-scale multicenter studies for the evaluation of AI-based models. This is due to two main reasons: First, it is crucial to incorporate a reproducible and representative group of the whole population, and not to neglect a specific gender or an ethical minority. This type of “ethical responsibility” (84) can only be addressed through large-scale multicenter studies and will ultimately lead to a reduction of existing biases for those complex predictions. Secondly, only data externally validated and further tested in prospective controlled trials will have the prognostic power to pave the way for a standardized risk classification tool. As with pharmaceutical products, some AI-algorithms are classified as a ‘medical device’ and are subjected to regulations by the Food and Drug Administration (FDA) of the European Medical Agency (EMA) [91]. Sometimes, FDA or EMA-approved algorithms pass through a verification of performance in clinical trials. However, in most cases for EMA approval and in some cases for FDA approval this step is skipped [92].

On a research level, there is a need for benchmarking datasets in atherosclerotic CVDs through a performance review in a similar way already performed in brain tumors [93], vertebrae labelling [94], or liver tumor segmentation [95]. This allows testing how well DL-models from different research groups are working in direct comparison to each other. In terms of data quality, a variety of current limitations need to be mentioned. When considering the fact that radiomic analysis relies on stable DL-based segmentation algorithms for large-scale analyses, the robustness of ground truth segmentations by human operators is crucial. However, several studies have highlighted the high inter-/intraobserver variability. Maroules et al. [96] investigated that even with an overall **κ** of 0.40 for high-risk plaque segmentations in general, they only achieved 0.15 < **κ** < 0.34 for positive remodelling and the Napkin-ring sign. Kolossváry et al. [97] also pointed out a considerable disagreement in LAP volumes. Therefore, there is the unmet need to build a valid ground truth for coronary plaques e.g. by using histopathological data from post-mortem studies analyzing plaque volume and composition and correlating those results with its volumetric data from CCTA.

Regarding the ground truth it should be reconsidered which manual segmentation technique is being applied. In nearly all of the current research applications a thresholding approach for non-calcified components is used in which the voxels between the inner and outer wall of the coronary vessel not appearing in the calcified range will be summed up to the non-calcified plaque volume [34,76]. This approach has been further investigated by Schlett et al. [58]. They compared two segmentation methods: a semi-automatic thresholding-based approach and a delineation of the low-attenuation plaque portion itself. It was shown that the diagnostic accuracy in comparison with histology was higher with the second method while the first method led to larger low-attenuation plaque areas (discriminative ability of CCTA tested for various thresholds: <30 HU: AUC 0.095 (p = 0.001), <60 HU: AUC 0.055 (p = 0.02), <90 HU: AUC 0.051 (p = 0.005)). They explained these results by the fact that the vessel wall itself consists of fibrotic tissue in which the voxels have overlapping HU-ranges with these plaques.

Further, the definition of HRP features should also be reviewed. To date, numerous definitions have been used with comparably good results. However, when radiomics should be trained for plaque type characterization, the discrepancies in the definitions themselves should be clarified in advance. Otherwise, the comparability and interpretation of different ML-model outputs will always be affected. In addition, and with regards to upraising new technologies and knowledge from previous studies, it would be necessary to reconsider which plaque features actually contribute to a high-risk plaque category. It could be analyzed if, for example, mixed plaques with an only very low calcified proportion < 1000 HU might be associated with a significantly higher risk for ACS than mixed plaques with a largely calcified portion. Further, structural properties obtained from radiomic analysis could also contribute new findings to risk stratification in the category of non-calcified/low-attenuation plaques.

Lastly, technical parameters during input data acquisition should be reviewed before training DL-models on coronary plaques. This affects natural restrictions by the CCTA-technique itself. On the one hand, blurring can lead to overestimation of calcification [98]. Therefore, the right windowing is crucial. On the other hand, partial volume effects can affect the attenuation of NCPs, leading to higher average attenuation values [99]. This is particularly relevant considering that the first low-attenuation plaque definitions were obtained several years ago with a larger slice thickness [28]. For this reason, partial volume effects could have had a much greater impact on mean attenuation values of individual voxels compared to modern CT scanners.

In the evaluation of DL-model outputs for assessing atherosclerotic CVDs there are three major considerations: First, the metrics for measuring output data should be treated with caution. Although there seems to be the current perception that a Dice coefficient > 0.90 is necessary for a good segmentation model, this metric is affected by the size of the input structure. When segmenting big structures such as the pericardium, the Dice coefficient usually shows excellent results around 0.93 [100]. However, Dice coefficients for different types of coronary plaque segmentation were mostly in the range between 0.58 and 0.73 [34,101]. This is crucial to assess when evaluating the quality of results.

Secondly, the level of detail used in plaque characterization should be reconsidered. In recent publications in the field of automated coronary plaque segmentations, a separation was only made between calcified and non-calcified plaques [34,76]. Further differentiations such as the distinction between calcified plaques and spotty calcification or the automatic segmentation of Napkin-ring signs have not been included so far. Even if the differentiation between low-attenuation plaques (< 30 HU) and non-calcified plaques has been partially made, it was based on a thresholding approach with the above-mentioned limitations.

Third, although great progress has been made in the automation of coronary plaque segmentations in recent years, an application with a truly completely fully-automated approach is not yet available. To handle large volumes of CCTA imaging data, automated segmentation and classification of coronary plaques based on the CCTA image alone would be desirable. However, until now human corrections are still necessary at various points in this pipeline. This involves corrections of the centerlines, exclusion of scans with insufficient quality, or cropping of start- and end points of plaques on a coronary centerline to get the volumetric data of plaque components.

Therefore, there are four major steps for future research in this field. First, as already described in detail, plaque definitions and segmentation techniques for the construction of the ground truth should be re-evaluated. Secondly, the influence of technical scan parameters such as slice thickness on radiomic signatures should be systematically evaluated through technical studies. This applies in particular to the improved spatial resolution of CT technology with lower slice thickness, such as the improved resolution of the photon counting detector CT (PCCT). Initial clinical studies on PCCT have shown both reduced blooming artefacts of calcium and better visualization of non-calcified plaques in terms of lipid-rich or fibrotic plaque components [102]. In this context, the applicability of existing AI-based imaging markers such as FAI, the FRP or radiomics signatures of HRP features should be reviewed on PCCT scans. In terms of FAI, biomarkers based on attenuation values in/around coronary arteries would be expected to lead to lower HU values on PCCT as fewer and smaller voxels would be affected by partial volume effects. In particular for AI-imaging markers using the full range of radiomics for plaque characterization, future studies would need to investigate to what extent previous radiomic signatures can be reproduced. Further, new research possibilities using PCCT to develop new AI-based biomarkers for coronary plaque characterization should be considered. Given the increased resolution of PCCT and the fact that radiomics measure spatial relationships between voxels, there is the potential to develop newer and improved biomarkers by revealing new spatial relationships in tissue leading to an increase in the long-term prognostic value and risk evaluation of coronary artery disease.

A third major area for future research projects are large multicenter studies, which should include a patient population that is large in terms of numbers and able to reflect the detailed association with plaque-specific risk factors. These criteria are currently combined in the large Oxford Risk Factors And Non-invasive imaging (ORFAN) study, a multicenter study cohort that covers both prospective and retrospective CT scans of the chest, abdomen and pelvis. With currently over 28 international and 17 UK sites, this study will cover more than 200,000 CCTA scans including risk factors and outcome data from 18 to 99-year-old patients over a median follow-up period of up to 10 years. With access to national registries such as the NHS England and the National Institute for Cardiovascular Outcome Research (NICOR) further datasets with long term clinical outcomes will be available. This will offer a valuable platform linking imaging data with clinical outcomes and providing access to the wider research community to develop and test new imaging biomarkers against actual cardiac events.

The fourth and last point addresses the economic and legal aspects of such AI-based applications. Even if larger and more detailed studies of AI-based models are constantly being developed in the field of coronary plaque imaging, the benefits of those will only be fully exploited if they are successfully established in clinical practice. For this, a rigorous evaluation under the aspects of accountability of AI as well as improvements in clinical outcome and cost reduction is essential.

## 6 Conclusion

In conclusion, standardization is the key to developing meaningful AI-based models. While sophisticated tools are being developed in research, there is the need for standardized applications for measuring coronary inflammation in clinical practice and their regulatory clearance with the appropriate label. These tools are available for measurements of coronary inflammation (e.g. FAI-Score interpreted on age and sex-specific nomograms) and risk prediction tools which integrate coronary inflammation, risk factors, and information related to the plaque distribution in coronary trees. With further analyses of large-scale outcome cohorts, these models are continuously recalibrated and adjusted, making risk prediction more accurate and risk management more efficient. It is expected that these approaches will dominate risk-guided management in primary and secondary prevention in the near future.

## Funding

CA is funded by the British Heart Foundation (CH/F/21/90009 and RG/F/21/110040), the EU Research and Innovation Action MAESTRIA (Grant agreement ID: 965286), the NIHR Oxford Biomedical Research Centre (Cardiac and Imaging themes), and Oxford British Heart Foundation Oxford Centre of Research Excellence (RG/18/3/34214). LK is supported by the Rhodes Trust.

## Declaration of competing interest

The authors declare the following financial interests/personal relationships which may be considered as potential competing interests: CA is founder, shareholder and director of Caristo Diagnostics Ltd, a CT-image analysis company. CA is the inventor of patents US10695023B2, US11393137B2, GB2018/1818049.7, GR20180100490, and GR20180100510, these are licensed to Caristo Diagnostics. CA has a leadership role in British Atherosclerosis Society and participates in several European commission Marie Curie Panels, and has received honoraria from Amarin, Covance and Slience Therapeutics.
